# The Efficiency of Bacterial Vaccines on Mortality during the ‘Spanish’ Influenza Pandemic of 1918–19

**DOI:** 10.1093/shm/hkad012

**Published:** 2023-05-08

**Authors:** David T Roth

**Affiliations:** School of History, Australian National University, Canberra, ACT 2600, Australia

**Keywords:** bacterial vaccines, viruses, ‘Spanish’ influenza, pandemic

## Abstract

The worldwide ‘Spanish’ influenza pandemic of 1918–19, which extended into the 1920s, infected more than a third of the world’s population and killed an estimated 50–100 million people, more than the civilian and military casualties of World War I. Present-day medical scholars, journalists, and other commentators have often ignored, downplayed or treated with scepticism the role of bacterial vaccines in reducing mortality during the pandemic. There have been repeated claims in this century that these vaccines were ‘useless’, ‘concocted’, and possibly harmful. Focussing on the Australian scene, I show that bacterial vaccines from reputable sources did indeed reduce mortality, perhaps to a greater extent in some cases than modern anti-viral influenza vaccines.

A 2011 guest post on the *History of Vaccines* blog, sponsored by the College of Physicians of Philadelphia, claims:

In 1918 influenza had neither treatment nor an effective vaccine. Indeed, most experts at the time thought that a bacterium caused influenza, rather than a virus. And though vaccines existed for several other diseases, and a few useless and possibly harmful anti-flu vaccines were concocted, an effective influenza vaccine was decades away.^1^

This critique exemplifies one of the chief arguments of modern day critics about the inefficiency of the bacterial vaccines used in the 1918–19 pandemic, namely that the developers were unaware that the bacterial antigens in the vaccines could not affect the influenza virus.^2^ As I show here, this argument ignores the significant role of secondary bacterial infections. Doctors at the time were well aware that such infections were a major contributing cause to the deaths of influenza patients. Other present-day critics downplay, deny or ignore apparently successful trials because they claim that the trials were poorly conducted according to present-day standards. I argue that such commentary gives a misleading picture of the state of medical knowledge at the period, the understanding of the immediate causes of death, the methodology of vaccine trials and the effectiveness of these pharmaceutical interventions. To refute these claims, I first discuss what we know of the state of knowledge about viruses, vaccines and causes of death in 1918–19. A number of evidently successful vaccine trials during the pandemic are then examined. The results suggest that the effectiveness of these vaccines against mortality was comparable with that of some modern day influenza vaccines. It follows that the health authorities and medical experts of the day were not as uninformed or naïve as some modern critics suggest.

## Criticisms of the 1918–19 Bacterial Vaccines

There are several common threads in contemporary criticisms of the pandemic vaccines and studies of their effectiveness. Firstly, as seen in the above *History of Vaccines* post,^3^ it is argued that bacterial vaccines could have had no effect because the medical workers of the time did not realise that the causative agent of influenza was a virus, and not a bacterium.

Miles Ott and his colleagues state that the two bacterial vaccines used in Minnesota were not effective as they did not contain the influenza virus.^4^ The ‘tremendous enthusiasm’ for using vaccines exceeded by far the scientific knowledge and technological capacity of the day, according to Jason Schwartz.^5^ In a 2014 *Lancet Respiratory Medicine* article, Hannah Cagney claims that the vaccines were ‘completely ineffective’ because they were based on ‘bacteriological theories’.^6^ Martin Gorsky and his associates argue that because contemporary medical authorities did not realise that influenza was a virus, the efficacy of the vaccines was limited, ‘except possibly against secondary infections’.^7^

Some studies ignore, downplay or deny the use of bacterial vaccines. The possible effects of mass vaccination on mortality are not mentioned in a study by Peter Caley and his co-authors on the role of social distancing in saving lives during the pandemic in Sydney, Australia. Perhaps half of the population of New South Wales (NSW) had been inoculated with a state-provided bacterial vaccine against influenza by May 1919.^8^ Lori Loeb, in her study of responses to influenza in Britain from 1889 to 1919, does not discuss vaccines although many military persons were inoculated.^9^ There were no vaccines to help the pandemic’s victims in California, according to Diane North.^10^ Sandra Tomkins claims incorrectly that there were ‘no vaccines or specific cures for influenza’ in 1918–19. She writes that effective vaccines had to await the discovery of its virus in 1933.^11^ Implicit in her claim is the assumption that the immediate causes of death were not bacterial.

Another thread of criticism is the claim that bacterial vaccines were probably ineffective because they could not have contained a sufficient spectrum of the strains or serotypes of virulent bacteria currently prevalent in persons diagnosed with influenza. The use of current variants of bacteria is important because immunity to a given strain or serotype of the same species may not mean immunity to another strain or serotype.^12^ As Dennis Shanks writes: ‘It is also difficult to see how such a mixed [bacterial] vaccine could generate an antibacterial immune response’.^13^ John Oxford and Douglas Gill maintain that no-one knew at the time that there were multiple serotypes of *Pneumococci*.^14^ We now know, for example, that *Streptococcus pneumoniae*, one of the components of the mixed-bacteria vaccines, has more than 90 serotypes.^15^ American virologist Stanley Plotkin expressed a similar view in the Philadelphia College of Physicians blog in 2018.^16^ A related approach is the use of loaded and emotive terms for the vaccines, their development, and their manufacture. Vaccines were said to have been ‘crude’, or in some cases ‘witches’ brews of injectable fluids’.^17^

A third line of criticism is that the studies of vaccine outcomes at the period were fundamentally unsound because they did not follow present-day standards for evaluation of medical trials.^18^ This means that reports of successes during 1918 and 1919 meet with deprecating or sceptical commentary. The methodological weaknesses of the 1918–19 studies include selection bias, unequal exposure to risk and unsatisfactory reporting of outcomes.^19^ Other deficiencies were the timing of vaccinations with respect to waves of infection and failure to account for confounding factors such as differing treatment regimes.^20^ John Eyler claims that better controlled studies, where risks of infection were more equalised between inoculated and uninoculated subjects, were less likely to conclude that the vaccine was effective. But I agree nevertheless with Eyler’s view that ‘[s]uch retrospective methodological criticism is unfair and ahistorical’. The criticism is unfair because careful testing was impossible because physicians were inexperienced in the conduct of trials and health resources were ‘stretched to the limit’. It is ahistorical because physicians were judged according to standards which did not then exist.^21^

## The State of Vaccine Technology in 1918–19

Vaccination was a well-established technology by 1918. Indeed it had been practised for hundreds of years, the best known example being Edward Jenner’s successful inoculations in the 18th century against the smallpox virus using preparations of the much milder cowpox.^22^ Jenner’s program was pragmatic and not based on theory. Its widespread adoption and its endorsement by the British parliament indicate that his vaccine was reasonably effective.^23^ Although the germ theory of disease had been established since the mid-nineteenth century, Robert Koch, a German physician, became the first to link a specific bacterium to a specific disease in 1876. Koch’s use of solid culture media for growing bacteria and microscopy were ground-breaking developments.^24^ Although Koch’s reputation as a medical scientist was adversely affected by his unsuccessful tuberculosis vaccine, this vaccine was nevertheless developed into a useful diagnostic aid in the 20th century.^25^ Serums derived from the blood of animals immune to the targeted disease could also confer immunity. Emil von Behring and Shihasaburo Kitasato, working at Koch’s Institute for Hygiene in Berlin, developed the first serums for diphtheria and tetanus.^26^

At around the time of Koch’s ground-breaking work, Louis Pasteur, an eminent French scientist, was experimenting with transmission of bacterial infections among poultry via inoculation. He and his co-researchers found that killed or weakened (attenuated) bacteria did not kill the host and conferred immunity.^27^ Pasteur’s vaccines for anthrax in livestock and fowl cholera were highly successful.^28^ The human cholera vaccine, developed by Waldemar Haffkine at Pasteur’s Institute in Paris, proved to be effective in the Indian epidemics of the 1890s.^29^ Pasteur also applied the attenuation technique for the preparation of an effective vaccine against viral rabies. Stock serums against *Pneumococcus* bacteria appeared in the H. K. Mulford catalogue in 1895.^30^ The British bacteriologist Almroth Wright ran the first trial of a whole-cell heat-killed *Pneumococcus* vaccine from 1911 to 1912. While earlier trials among South African miners did not produce significant results, a trial in late 1912 with a higher concentration of bacteria reduced the death rate by 40 to 50 per cent. Pneumococcal vaccines using a mixture of serotypes were deployed early in the pandemic with ‘Spanish’ influenza patients at United States Army Camps Wheeler and Upton. They apparently reduced mortality markedly.^31^ Vaccination was by no means a new and untried technology in 1918. And anti-bacterial vaccines still play a role up to the present day in reducing secondary infections.^32^ So it is puzzling that there has been so little recognition that the bacterial vaccines of 1918–19 might have played a similar role.

As noted earlier, critics have stressed the methodological weaknesses (by modern standards) of some influenza vaccine trials. It does not follow that researchers from the late 19th century were necessarily uninformed about the latest statistical methods and the value of avoiding selection bias. The Pasteur Institute employed statistical techniques extensively in their studies of tetanus, typhoid, gangrene, typhus and staphylococci in the decades preceding World War I. In 1898, Johannes Fibiger, a Danish physician, conducted a genuine randomised trial of an anti-diphtheria serum.^33^ But Ronald Fisher, an eminent British statistician, did not publish the theoretical statistical advantages in experimental design of random allocation until 1925 in his definitive work *Statistical Methods for Research Workers*.^34^ Researchers from the 19th century had long advocated ‘alternate allocation’ to treatment comparison groups as a means to avoid selection bias.^35^

## The Understanding of Viruses as of 1918–19

In 1881 Pasteur wrote that ‘the virus is a microbial parasite, which may be multiplied by cultivation outside the body of an animal’.^36^ The first understandings of viruses, as now we know them, were obtained via a filter developed by in Pasteur’s laboratory by Charles Chamberland in 1884, which was used to remove bacteria from liquids. By the 1900s the Chamberland-Pasteur filter was in widespread use to deliver potable water.^37^ This device allowed the Russian botanist Dmitri Iwanowski to determine in 1892 that the disease-causing agent for tobacco mosaic disease was not bacterial.^38^ Martinus Beijerinck, a Dutch botanist, proposed that these filter-passing ‘microbes’, which were invisible to the microscopes of the day, should be called viruses. By 1906, 18 filter-passing agents associated with plant, animal and human diseases had been identified.^39^ The term ‘virus’ (in Pasteur’s sense) had been widely used in medical articles in the press well before the influenza pandemic.^40^

In October 1918 John Bradford, Ernest Bashford and J. A. Wilson, medical researchers for the British Army, claimed that they were able to culture a filter-passing virus derived from the blood of patients with acute and severe influenza. This finding was reported in the *British Medical Journal* (*BMJ*) on 1 February, 1919 and was widely reported in the Australian press as the discovery of ‘the influenza virus’ within a week.^41^ This speed of dissemination suggests that Australia was well in touch with recent scientific developments. The term ‘influenza virus’ was frequently used in Australian newspaper articles after the *BMJ* report.^42^ But Bradford et al.’s claims of an isolatable disease agent were rebutted on methodological grounds by the end of the year. J. A. Arkwright examined their filtered preparations of sputum from influenza patients and found that they were contaminated with disease-causing bacteria, and that control samples appeared identical to the filtered samples. Although animals inoculated by Bradford’s team with the filtered samples did show signs of disease, Arkwright contended that the contamination made it impossible to prove that their sickness was caused by influenza. In the same issue of the *BMJ*, both Bradford and Wilson accepted that their claims were not proven.^43^ Arkwright suggests that the experimental failings of Bradford and his colleagues were likely due to rushed work under wartime conditions.^44^ Evidence for an isolatable influenza virus in humans was not generally accepted until the work of Wilson Smith and his colleagues in 1933.^45^ Yet it seems clear that researchers in 1919 understood that viruses were disease causing agents which could not be observed in the microscopes of the time. W. G. Armstrong, an Australian researcher, postulated that the agent was a ‘minute organism’. J. Rose Bradford, a British researcher, had claimed that the actual agent was a bacillus.^46^ Researchers had indeed isolated filter-passing agents for other conditions, as noted above, despite the problematic work of Bradford et al in the case of influenza.

## The Causes of Death

During the ‘Russian’ influenza of the 1890s, Richard Pfeiffer, a German bacteriologist, claimed that *Bacillus influenzae*, which he had found in infected patients, was the actual cause of disease and death. This bacterium was later called Pfeiffer’s bacillus or *Haemophilus influenzae.* But by late 1918 this association had been discredited. Although initial trials in 1918 with single *H. influenzae* vaccines had been reported as successful, other researchers had had difficulty in isolating the bacillus from influenza patients. The most common organisms found were *Pneumococci* or *Streptococci.*^47^ Taking a precautionary approach, physicians lost confidence in *H. influenzae* vaccines and moved to mixed-bacteria preparations. Some omitted *H. influenzae* altogether.^48^ Nevertheless this bacterium is still seen as a cause of pneumonia. According to Eva Heinz, 21st century studies of the prevalence of the Hib type of this bacteria (then the majority type), showed that Hib vaccines had almost eradicated this form of pneumonia in some countries by the end of the 20th century.^49^

Early in the pandemic, physicians became aware that influenza itself was not necessarily the immediate cause of death. They did recognise that it was the primary or underlying cause of pandemic deaths; that is to say that these deaths would not otherwise have occurred without this initial infection. But published data from the period suggest that the immediate cause of the great majority of deaths was a secondary haemorrhagic bacterial infection of the lungs.^50^ Milton Winternitz and his associates, writing in 1920, suggested that the ‘unknown etiological agent in influenza’ injured the lungs, so that bacteria resident in the body could initiate infections such as pneumonia.^51^ Indeed, the official, medical and lay terminology for the disease at the time was ‘pneumonic influenza’.^52^ Swabs from living and deceased patients revealed the presence of a range of species of bacteria thought to be associated with fatal cases of pneumonia, including strains of *Pneumococci*, *Streptococci*, *Staphylococci* and *Diplococci*.^53^ These microbes were known at the time to be present in the respiratory tracts of healthy persons.^54^ Physicians reasoned that influenza infection had weakened the body’s defences, in particular, the lining of the lung. Hence these bacteria, in killed or weakened formulations, were seen as eligible candidates for effective vaccines, even though the medical profession suspected that these bacteria were not the underlying or primary causes of death and illness.

## The 1918–19 Studies of Mixed Bacteria Vaccines

There were apparent successes with mixed vaccines. Edward Rosenow, a pulmonary specialist based in Rochester, Minnesota, formulated a mixture of killed pneumococci, haemolytic streptococci, staphylococci and Pfeiffer’s bacillus. When he was convinced that the vaccine was safe and effective in small scale trials, it was distributed by the Mayo Foundation to physicians, hospitals and other institutions within a 320 kilometre radius. Three doses were given. Rosenow found that the mortality rate in Minnesota for the unvaccinated control patients was about four times greater than the rate for the fully vaccinated.^55^ But George McCoy, Director of the Hygienic Laboratory of the United States Public Health Service, and his associates concluded from a trial in a single state insane asylum that Rosenow’s vaccine was a ‘failure’. Eyler contrasts the superior methodology of McCoy’s study with respect to equalisation of risk as compared to the general methods of other studies of the time.^56^ Yet a 2009 metastudy of pandemic vaccine studies by Yu-Wen Chien and her colleagues found that Rosenow’s vaccine was protective, as I discuss later.^57^

A seemingly successful mixed vaccine, however, broadly similar in composition to Rosenow’s and containing Pfeiffer’s bacillus, was developed by the Australian government owned Commonwealth Serum Laboratories (CSL) in Melbourne in late 1918.^58^ CSL had been set up in 1916 to address wartime shortages of vaccines and other medical supplies.^59^ It is clear from the detailed description by its first director, W. J. Penfold, that the vaccine’s preparation used the most advanced technology of the day, for example in the painstaking measures taken to avoid contamination of the culture media for the growth of bacteria derived from patient samples.^60^ It could not be described as a ‘witches brew’.^61^ Thomas Cherry, a noted Australian bacteriologist, found that trials of the CSL vaccine in isolation hospitals in Melbourne from late January to the middle of March 1919 showed a 3:1 reduction in the overall death rate for inoculated versus uninoculated patients (4.0 per cent as against 13.8 per cent).^62^

Positive results were also claimed for the CSL vaccine when it was used in the Samoan relief expedition of December 1918, led by Surgeon Lieutenant Francis Grey. Responding to an urgent call for help in November, a medical team with vaccine supplies was sent from Australia in quick time. While en route, the vaccine was also issued to the crew of the relief ship, *HMAS Encounter*. But when the expedition arrived, about a quarter of the indigenous population had already died and, according to Shanks, the epidemic was waning. Grey noted that the case mortality rate for resident Europeans was only two per cent. Shanks claims that no actual figures were produced for the CSL vaccine’s effectiveness in Samoa. I have already mentioned his scepticism about the usefulness of mixed bacterial vaccines. Yet by the time the *Encounter’s* crew had returned to Sydney, at the height of the first wave of the pandemic in that city in March 1919, 22 per cent had been ill, but none of them contracted pneumonia or died.^63^ During the first half of 1919, Gray observed that of the 2,875 sailors inoculated at the Williamstown naval base in Melbourne, only 345 were infected and none died.^64^

Significant reductions in mortality were observed in New South Wales public hospitals with the CSL vaccine or with the similar ‘state’ vaccine, which incorporated some serums supplied by CSL. Among 13,000 patients treated for influenza, mortality among the unvaccinated was 16.5 per cent, while the mortality for the vaccinated was 10.7 per cent for all persons who had received at least one inoculation.^65^ A study of 1,401 patients in Melbourne public isolation hospitals admitted between the end of January 1919 and the middle of March showed that the overall mortality of unvaccinated persons was more than twice the mortality of the vaccinated (9.3 per cent versus 3.9 per cent). Patients hospitalised in the early days of infection had a much lower death rate, whether vaccinated or not.^66^

With respect to the above-mentioned critiques about the use of relevant bacterial variants in the vaccines, CSL made a determined effort to secure as many strains as possible from current or deceased patients. For example, 170 cultures of pneumococcus-like organisms from 97 different sources were used in the vaccines, while 27 cultures of streptococcus from 20 sources were also included. Swabs were taken mainly from Victorian patients, with some provided from the North Head quarantine station in Sydney. It should be noted that CSL found significant numbers of Pfeiffer’s bacillus (*H. influenzae*) in the lungs and that this bacterium was included in the vaccine.^67^

For indigenous Australians living in remoter areas, we can assume that vaccines and other medical interventions came late or not at all. In Queensland, about 30 per cent of all reported influenza deaths were among Aboriginal people, approximately two per cent of the estimated Indigenous population in that state. Gordon Briscoe attributes the high death rate to lack of immunity among people in remote settlements. Indigenous people from these communities were forced to move to makeshift hospital ‘camps’ for treatment where they received little or no care, poor nutrition, were exposed to other infections and had minimal shelter.^68^ We can infer from this poor standard of care that Aboriginal communities, in Queensland at least, were likely not included in influenza vaccination campaigns.^69^ From the chaos which Briscoe describes when many deaths occurred on the same day, it is probable that the death rate was significantly undercounted.^70^ Indigenous peoples around the world suffered disproportionate death rates as compared to Europeans, especially among young adults.^71^ In some Indigenous communities in Alaska the mortality rate was greater than 80 per cent. Children were the only survivors in some Arctic villages.^72^

There were other evident successes for mixed vaccines in Canada and Britain. A vaccine was prepared by Fred Cadham in 1919 for the treatment of soldiers suffering from influenza at the Tuxedo Medical Hospital in Winnipeg, Canada. Cadham found that no military patient died who had received two doses, whereas 17 died among the 238 uninoculated patients. The average hospital stay was twice as long for the unvaccinated. About a quarter of the civilian populations in Manitoba and Saskatchewan who were inoculated received Cadham’s vaccine, while the remainder were given the Rosenow vaccine. Among the approximately 53,000 vaccinated civilians in these provinces, the death rate for the twice inoculated was about half that for those vaccinated once only, and about a seventh of the mortality rate for notified, unvaccinated patients. Cadham did not mention any attempt to match the civilian control group with the vaccinated subjects.^73^ A mixed vaccine recommended by William Leishman, Director of Pathology at the War Office in London, was given to 15,624 military personnel, whereas 43,520 were not vaccinated. The death rate in the control group was about 12 times the death rate in the vaccinated group. Although the test was poorly controlled by modern standards, both groups were similar in age and health.^74^ As with the CSL vaccines, the bacteria used in these Canadian and British vaccines were all derived from influenza patients within the general area of the manufacturing laboratories.

## For-Profit Vaccines

There was substantial ‘boosting’ of for-profit bacterial vaccines at this period in the USA and Australia. For example, there were many advertisements for proprietary vaccines and influenza ‘cures’ in Australian newspapers. Donald Ross and Company of Sydney, which also marketed veterinary vaccines, offered a ‘Pneumococcic-Influenzal’ vaccine.^75^ Australian doctors were advised in February 1919 that ample supplies of vaccine were available from Elliott Brothers Surgical Department in Sydney.^76^ An ‘unlimited quantity’ was available from Francois Ray’s Vaccine Institute in Sydney. Ray, a French bacteriologist, was probably a reputable source, having worked with Pasteur in Paris. Arriving in Sydney in 1911 as the Pasteur Institute’s representative in Australia, he founded his own institute sometime before 1918.^77^ The Biological Institute of Australasia, a private company, offered a ‘high concentration of specific pneumococci organisms’ in its vaccine, costing £1 per tube of double inoculations, not including the doctor’s profit margin.^78^ Some anti-vaccination voices at the period accused private suppliers of reaping a ‘bonanza’ from a credulous public.^79^ Presumably these more expensive offerings, which would likely have attracted the anxious and wealthy, might have been seriously undercut by the free CSL vaccine offered in the mass vaccination drives of local councils in Victoria and New South Wales which were co-ordinated by the public health authorities. These campaigns were substantially assisted by thousands of unpaid volunteers.^80^

In the USA, private drug companies promoted vaccines which had been in stock for some years, including preventatives against the common cold. If these older vaccines did perchance contain killed bacteria associated with the secondary infections of the pandemic, they were unlikely to be effective because they probably were not up-to-date with the strains and serotypes present in 1918 due to evolutionary processes over time. We know now that pathogens, including pneumonia bacteria, can rapidly alter their strain dynamics and serotype composition in response to genotype changes in their hosts as the latter develop resistance.^81^

According to Eyler, there was also a great deal of ‘price-gouging’ by commercial interests and ‘hucksterism’ by individual medical practitioners. Max Exner, author of several books on sexuality, issued ‘glowing testimonials’ to his own successful use of an omnibus vaccine for influenza, blood poisoning and pneumonia. This vaccine had been developed six years earlier by Ellis Bonime at the Polyclinic Medical School in New York. Its contents were undisclosed.^82^ G. H. Sherman of Detroit marketed a mixed vaccine which claimed to ‘abort’ colds, influenza and pneumonia.^83^ The privately-owned Cutter Laboratories of Chicago declared that their mixed vaccine gave ‘complete protection’.^84^ While it is possible, as may have been the case with Ray’s vaccine in Sydney, that some of these commercial vaccines were effective, their providers had an interest in advertising their own successes. There is minimal independent evidence available of the effectiveness of their offerings.^85^

## Vaccine Effectiveness and Efficacy: What They Mean and How They Are Measured?

In order to make a comparison of the effectiveness of the 1918–19 vaccines in reducing mortality with the effectiveness of modern anti-viral influenza vaccines, a standard of measurement is required. Here I need to explain the technical terms vaccine efficacy and vaccine efficiency. According to the World Health Organization (the WHO), vaccine efficacy is a measure of the outcome of a controlled clinical trial where the unvaccinated group receives a placebo. Ideally, the ‘gold standard’ is a double-blind study, where neither the participants nor the inoculators know if the inoculation is the actual vaccine, and the selection of subjects is randomised. Vaccine efficiency, on the other hand, is a measure of the outcome in the ‘real world’, where the researchers have no control over who is selected for vaccination.^86^ Because the 1918–19 vaccine trials were poorly controlled or not controlled by modern standards, it seems that ‘efficiency’ is the more appropriate term to use for these studies.

Nevertheless Chien and her colleagues preferred to use the term ‘efficacy’ in their 2009 metastudy. They conceded that none of the thirteen military and civilian studies they selected were double-blind randomised studies. But they evidently believed that the statistical techniques they used could compensate for the methodological deficiencies in the selected studies, allowing them to speak of ‘efficacy’. Over 460 studies were not selected due to low sample sizes or insufficient information. Adjusting for publication bias (i.e., taking unpublished results into account), and confounding factors, Chien and her co-authors estimated that the efficacy for the remaining thirteen studies was about 34 per cent in preventing pneumonia and 42 per cent for case fatality.^87^ These data might be roughly compared with the estimated 30 per cent vaccine efficacy against mortality during the 1989–90 influenza epidemic in England.^88^

Table 1 shows my estimates for the efficiency of selected 1918–19 vaccines, together with published estimates for these vaccines by Chien et al. I also show estimates for a modern influenza vaccine and COVID. For my calculations, I used standard formulae endorsed by the WHO for measuring either efficiency or efficacy of vaccines. These formulae, which have been in use since 1915,^89^ are used to estimate the value of vaccines with respect to either death or infection rates.^90^

**Table 1.** Vaccine efficiencies and efficacies with respect to mortality

Study	Vaccine efficiency or efficacy (%)
Armstrong, 1920, CSL vaccine efficiency, New South Wales hospitals, 1 vaccination.^91^ (my calculation)	32.7
Armstrong, 1920, CSL vaccine efficiency, New South Wales hospitals, 2 vaccinations.^92^ (my calculation)	50.3
Cherry, 1920, CSL vaccine efficiency – Melbourne hospitals^93^ (my calculation)	71.0
Chien et al., 2009, metastudy of 1918–19 vaccines (overall efficacy, civilian)^94^	42.0
Chien et al., 2009, metastudy of 1918–19 vaccines (overall efficacy, military)^95^	70.0
Ahmed et al., 1995, 1995–89–90 influenza deaths, England (efficacy)^96^	41.0
Nation et al., 2021, Australian hospitals influenza deaths 2010–17 (effectiveness)^97^	31.0
Jablonska et al. 2021 – COVID deaths - Europe & Israel (Pfizer) (overall efficacy)^98^	72.0


$$\begin{array}{l} VE\left(\%\right)=\left(ARU-ARV\right)\;\times \;100/ARU \end{array}$$

Where ARU = the attack rate in the unvaccinated population and ARV = the attack rate in the vaccinated population. The attack rate for mortality is the percentage of an at-risk population that dies from the disease.

-OR -

VE (%) = (1 – RR) × 100 where RR = relative risk, the probability of an event occurring in the treated group divided by the probability of the event in the untreated group.

The measures of efficiency and efficacy with respect to mortality in Table 1 suggest that the 1918–19 bacterial vaccines were roughly comparable with modern influenza vaccines.

## Conclusions

Claims that physicians and researchers in 1918–19 had little or no knowledge either of the nature of viruses or of the immediate causes of death during the pandemic are not supported by the evidence. It is clear that many researchers had an understanding of viruses as causative factors for disease and death by 1918. They also understood that the immediate causes of the great majority of influenza deaths were bacterial secondary infections. While researchers could not establish a specific bacterium to be the cause of death, vaccine makers took a conservative approach, using a range of species and strains found in significant numbers in patients. Oxford and Gill’s claim that no one knew at the time that *Pneumococcus* had many serotypes is correct, but seems unfair in this context and beside the point in judging the effectiveness of vaccination. Physicians certainly knew that *Pneumococcus* and other bacteria had several strains and indeed used a range of types derived from current patients in vaccines. It is also clear that vaccine technology was well established and reasonably successful by 1918. Penfold’s detailed account of the preparation of the CSL vaccine, for example, with its frequent references to international practices and research, undermines claims that the preparation of this vaccine was based on ignorance and incompetence rather than the latest science.^99^

Despite the methodological weaknesses of the 1918–19 studies as seen from present-day perspectives, the evidence suggests that the mixed bacterial vaccines provided by reputable sources during the pandemic were reasonably effective in preventing mortality. Substantial differences in mortality between inoculated and uninoculated in a variety of jurisdictions cannot simply be dismissed en bloc as failures to conform to modern standards of medical trials. The health authorities and medical experts of the day were not uninformed, naïve or hapless with respect to pharmaceutical interventions for influenza, or the causes of mortality. As Michael Bresalier notes: ‘Retrospective judgments of the failure of laboratory medicine to control influenza not only ignore the state of contemporary scientific knowledge and practices, but also how experts responded to the challenges’.^100^

Despite a wave of historiography about the pandemic in this century which has critically re-examined or revealed a mass of evidence,^101^ unfair sceptical commentary about the influenza vaccines, and the alleged misunderstandings of medical experts of the time, persist.^102^ It is curious that such critiques have not been focussed on other pre-1919 vaccines (viral and bacterial), such as the pneumonia vaccines discussed earlier.^103^ For these vaccines, their theoretical underpinnings, their trials and their standards of preparation, also did not meet modern standards. This forgetting may perhaps be explained when it is understood that most of today’s effective vaccines began development during the ‘Golden Age’ of the 1950s. The most famous example was the Salk and Sabin vaccines for polio, available in the early 1960s.^104^ It is likely that the sheer bulk of more recent scientific knowledge, and the successes of later vaccines, have induced commentators to view the pandemic vaccines, and their developers, through a modern lens, rather than on their own terms. Yet anti-bacterial vaccines still have a role to play in influenza pandemics in reducing secondary infections.^105^

## Footnotes

1 History of Vaccines, ‘Influenza Pandemics – A Brief History’, accessed January 8, 2023, https://www.vaccinestoday.eu/stories/influenza-pandemics-a-brief-history/

2 Antigens, the active components of vaccines, are molecules which trigger immune responses in the body.

3 History of Vaccines, ‘Influenza Pandemics – A Brief History’, accessed January 8, 2023, https://www.vaccinestoday.eu/stories/influenza-pandemics-a-brief-history/

4 Miles Ott, Shelly F. Shaw, Richard N. Danila, and Ruth Lynfield, ‘Lessons Learned from the 1918–1919 Influenza Pandemic in Minneapolis and St. Paul, Minnesota’, *Public Health Reports*, 2007, 122, , 803–10, 807.

5 Jason L. Schwartz, ‘The Spanish Flu, Epidemics, and the Turn to Biomedical Responses’, *American Journal of Public Health*, 2018, 108, 1455–58.

6 Hannah Cagney, ‘America’s Response to the 1918 Influenza Epidemic’, *Lancet Respiratory Medicine*, 2014, 2, 792–93.

7 Martin Gorsky, Bernard Harris, Patricia Marsh, and Ida Milne, ‘The 1918/1919 Influenza Pandemic & COVID-19 in Ireland and the UK’, *Historical Social Research, Supplement*, 2021, , 193–226, 210.

8 Peter Caley, David J. Philp, and Kevin McCracken, ‘Quantifying Social Distancing Arising from Pandemic Influenza’, *Journal of The Royal Society Interface*, 2008, 5, , 631–39. For numbers inoculated in New South Wales, see W. G. Armstrong, *Report of the Director-General of Public Health to the Honourable Minister of Public Health on the Influenza Epidemic in New South Wales* in *Joint Volume of Papers Presented to the New South Wales Legislative Council and Legislative Assembly, First Session, 1920* (Sydney: Government Printer, 1920), 155.

9 Lori Loeb, ‘Beating the Flu: Orthodox and Commercial Responses to Influenza in Britain, 1889–1919’, *Social History of Medicine*, 2005, 18, 203–24.

10 Diane M. T. North, ‘California and the 1918–1920 Influenza Pandemic,’ *California History*, 2020, 97, 3–36.

11 Sandra M. Tomkins, ‘The Failure of Expertise: Public Health Policy in Britain during the 1918-19 Influenza Epidemic’, *Social History of Medicine*, 1992, 5, 435–54, 437. See also Ryan M. Alexander, ‘The Spanish Flu and the Sanitary Dictatorship: *Mexico’s Response to the 1918 Influenza Pandemic*’, *The Americas*, 2019, 76, 443–65, 454.

12 A strain is a genetic variant or subtype of an organism. The term serotype is used to describe bacteria or viruses of the same species that are antigenically different. In either case, different strains or serotypes may evoke different responses from the immune system.

13 G. Dennis Shanks, ‘The ‘Influenza’ Vaccine Used during the Samoan Pandemic of 1918,’ *Tropical Medicine and Infectious Disease*, 2018, 3, 17–23, 21.

14 John S. Oxford and Douglas Gill, ‘Vaccines from the Spanish Influenza as a Firm Foundation for New Developments’, *Human Vaccines & Immuno­therapeutics*, 2020, 16, 2051–2055, 2052.

15 Ioanna Christopoulou, Kenny Roose, Lorena Itatí Ibañez et al., ‘Influenza Vaccines to Control Influenza-Associated Bacterial Infection: Where Do We Stand?’ *Expert Review of Vaccines*, 2015, 14, 55–67, 56.

16 René F. Najera, ‘The 1918-1919 Influenza Pandemic and Vaccine Development’, accessed 30 May, 2022, https://historyofvaccines.org/blog/the-1918-19-spanish-influenza-pandemic-and-vaccine-development.

17 John M. Eyler, ‘The Fog of Research: Influenza Vaccine Trials during the 1918–19 Pandemic’, *Journal of the History of Medicine and Allied Sciences*, 2009, 64, 401–28, 405. Eyler’s reference to “witches’ brew” is apparently derived from J. R. Paul’s well-known critique of early polio vaccines – see J. R. Paul, *A History of Poliomyelitis* (New Haven, Connecticut: Yale University Press, 1971), 258.

18 Eyler, ‘The Fog of Research’, 416.

19 Eyler, ‘The Fog of Research’, 415.

20 Shanks, ‘The “Influenza” Vaccine Used during the Samoan Pandemic of 1918’, 20.

21 Eyler, ‘The Fog of Research’, 415–16.

22 S. Riedel, ‘Edward Jenner and the history of smallpox and vaccination’, *Baylor University Medical Center Proceedings*, 2005, 18, 21–25. Jenner was not the first person to use cowpox inoculations.

23 Jenner was not the only proponent of the vaccine. See Cary P. Gross and Kent A. Sepkowitz, ‘The Myth of the Medical Breakthrough: Smallpox, Vaccination, and Jenner Reconsidered’, *International Journal of Infectious Diseases*, 1998, 3, 54–60.

24 Steve M. Blevins and Michael S. Bronze, ‘Robert Koch and the ‘golden age’ of bacteriology’, *International Journal of Infectious Diseases*, 2010, 14, e744–e751, e745–e746.

25 For Koch’s tuberculosis vaccine, see Donald S. Burke, ‘Of Postulates and Peccadilloes: Robert Koch and Vaccine (tuberculin) Therapy for Tuberculosis’, *Vaccine*, 1993, 11, 795–804.

26 Emil van Behring and Shibasaburo Kitasato, ‘Ueber das Zustandekommen der Diphtherie-Immunität und der Tetanus-Immunität bei Thieren’, *Deutsche Medizinische Wochenschrift*, 1890, 16, 1113–1114. Von Behring won the Nobel Prize for Medicine in 1901 for developing the diphtheria vaccine.

27 Agnes Ullmann, ‘Pasteur-Koch: Distinctive Ways of Thinking about Infectious Diseases’, *American Society for Microbiology*, 2007, 2, 383–87, 385.

28 Peter C. B. Turnbull, ‘Anthrax Vaccines: Past, Present and Future,’ *Vaccine*, 1991, 9, 533–39.

29 Jean-Marc Cavaillon and Marcin F. Osuchowski, ‘COVID-19 and Earlier Pandemics, Sepsis, and Vaccines: A Historical Perspective’, *Journal of Intensive Medicine*, 2021, 1, 4–13.

30 J. D. Grabenstein and K. P. Klugman, ‘A Century of Pneumococcal Vaccination Research in Humans’, *Clinical Microbiology and Infection*, 18, 2012, Suppl 5, 16. The H. K. Mulford company was a pharmaceutical firm based in Philadelphia, USA.

31
*Ibid.*, 15, 16, 18; Russell L. Cecil and Henry F. Vaughan, ‘Results of Prophylactic Vaccination against Pneumonia at Camp Wheeler’, *Journal of Experimental Medicine*, 1919, 29, 457–83, 458.

32 Ravindra K. Gupta, Robert George and Jonathan S. Nguyen-Van-Tam, ‘Bacterial Pneumonia and Pandemic Influenza Planning,’ *Emerging Infectious Diseases*, 2008, 14, 1187–92.

33 Gabriel Gachelin, ‘The Construction of a Culture of Standardization at the Institut Pasteur (1885–1900)’, Chapter 4 in Christoph Gradmann and Jonathan Simon, eds, *Evaluating and Standardizing Therapeutic Agents, 1890-1950* (Houndsmills, England: Palgrave Macmillan, 2010), 80.

34 R. A. Fisher, *Statistical Methods for Research Workers*, 7th Edition (Edinburgh: Oliver and Boyd, 1938). First Edition 1925. For initial discussion of benefits of random allocation see Introductory, 7–9.

35 Iain Chalmers, Estela Dukan, Scott Podolsky and George Davey Smith, ‘The Advent of Fair Treatment Allocation Schedules in Clinical Trials during the 19th and Early 20th Centuries’, *Journal of the Royal Society of Medicine*, 2012, 105, 221–27, 223.

36 Patrick Berche, ‘Louis Pasteur, from Crystals of Life to Vaccination’, *Clinical Microbiology and Infection*, 2012, 18, 1–6; Jeffery K. Taubenberger, Johan V. Hultin and David M Morens, ‘Discovery and Characterization of the 1918 Pandemic Influenza Virus in Historical Context’, *Antiviral Therapy*, 2007, 12, 581–91, 584. For Pasteur’s usage of ‘virus’, see entry in *Oxford English Dictionary* (Medicine).

37 W. H. Horrocks, ‘On the Protection from Waterborne Disease Afforded by the Pasteur-Chamberland and Berkefeld Filters’, *British Medical Journal*, 1901, 1, 1471–72.

38 Dmitri Ivanowski, ‘Ueber die Mosaikkrankheit der Tabakspflanze’, *St Petersburg Academie Imperial Science Bulletin*, 1892, 35, 67–70. See also Milton Zaitlin, ‘The Discovery of the Causal Agent of the Tobacco Mosaic Disease’, in Shain-Dow Kung and Shang-Fa Yang, eds, *Discoveries in Plant Biology* (Singapore: World Scientific, 1998), 105–110.

39 Taubenberger et al., ‘Discovery and Characterization of the 1918 Pandemic Influenza Virus in Historical Context’, 585.

40 For example, ‘Vaccines and Serums’, *Northern Star* (Lismore, NSW), January 21, 1916, 3; ‘A Disease of Fowls’, *Northern Territory Times and Gazette* (Darwin, Northern Territory), January 5, 1912, 4.

41 John Rose Bradford, E. F. Bashford and J. A. Wilson, ‘THE FILTER-PASSING “ “VIRUS OF INFLUENZA”’, *Quarterly Journal of Medicine*, 1919, 12, 259–306, 260; John Rose Bradford, E. F. Bashford and J. A. Wilson, ‘Preliminary Report on the Presence of a “Filter Passing” Virus in Certain Diseases: With Especial Reference to Trench Fever, Influenza, and Nephritis’, *British Medical Journal*, 1919, 1, 127–28. For press reports in Australia, see for example ‘The Virus Found Possibly Important Medical Discovery. (Published in ‘The Times.’) London, February 5’, *Daily Herald* (Adelaide), February 8, 1919, 6.

42 For example, ‘Women Scientists. Leaders in Medical Research. Tracking Influenza Virus’, *Maitland Daily Mercury* (Maitland, NSW), April 2, 1923, 8.

43 J. A. Arkwright, ‘Criticism Of Certain Recent Claims To Have Discovered And Cultivated The Filter-Passing Virus Of Trench Fever And Of Influenza’, *British Medical Journal*, 1919, 2, 233–36; John Rose Bradford and J. A. Wilson, ‘Notes on Dr Arkwright’s Article’, *British Medical Journal*, 1919, 2, 236–37. This debate is summarised in Islay Shelbourne, ‘The Influenza Problem: Paradigms, a Pandemic, and the Search for Pfeiffer’s Bacillus’ (MA Thesis, Institute of Historical Research, 2021), 85–86.

44 Arkwright *Ibid.*, 236.

45 Wilson Smith, Christopher H. Andrewes and Patrick P. Laidlaw, ‘A Virus obtained from Influenza Patients,’ *Lancet*, 1933, 222, 66–68. See also Jeffrey K. Taubenberger and David M. Morens, ‘The 1918 Influenza Pandemic and its Legacy’, *Cold Spring Harbor Perspectives in Medicine*, 2020, 10, a038695 and John M. Eyler, ‘De Kruif’s Boast: Vaccine Trials and the Construction of a Virus’, *Bulletin of the History of Medicine*, 2006, 80, 409–438.

46 Armstrong, *Report of the Director-General of Public Health,* 200; ‘The Micro Organism. Isolated by J. Rose Bradford’, *West Australian* (Perth, WA), May 7, 1919, 8.

47 John M. Eyler, ‘The State of Science, Microbiology, and Vaccines Circa 1918’, *Public Health Reports*, 2010, 125, 27–36, 31–3.

48 Eyler, ‘The Fog of Research’, 404–406.

49 Eva Heinz, ‘The Return of Pfeiffer’s bacillus: Rising Incidence of Ampicillin Resistance in *Haemophilus influenzae*,’ *Microbial Genomics*, 2018, 4, 5–11, 5.

50 For an understanding of the immediate and primary/underlying causes of death, see Iwao M. Moriyama, ‘Development of the Present Concept of Cause of Death’, *American Journal of Public Health and the Nations Health*, 1956, 46, 436–41. For immediate causes, see John F. Brundage and G. Dennis Shanks, ‘Deaths from Bacterial Pneumonia during 1918–19 Influenza Pandemic,’ *Emerging Infectious Diseases*, 2008, 14, 1193–99. See also David M. Morens, Jeffery K. Taubenberger, and Anthony S. Fauci, ‘Predominant Role of Bacterial Pneumonia as a Cause of Death in Pandemic Influenza: Implications for Pandemic Influenza Preparedness,’ *Journal of Infectious Diseases*, 2008, 198, 962–70.

51 Milton C. Winternitz, Frank P. McNamara and Isabel M. Wason, *The Pathology of Influenza* (New Haven, CT: Yale University Press, 1920), 43.

52 Armstrong, *Report of the Director-General of Public Health,* 141. See also ‘Ravages all over World (Published in ‘*The Times*’) London, Monday,’ *Herald* (Melbourne), October 22, 1918, 7. Pneumonic Influenza. ‘History and Treatment,’ *Sydney Morning Herald*, March 7, 1919, 6; ‘Soldiers’ Letters’, *Windsor and Richmond* Gazette (Windsor, NSW), January 10, 1919, 10; Peter Hobbins, ‘The Pneumonic Influenza is Just Part of my Life’: Fostering Community Histories of the “Spanish” Influenza in Australia,’ Chapter 11 in Guy Beiner, ed., *Pandemic Re-Awakenings: The Forgotten and Unforgotten ‘Spanish Flu’ of 1918-1919* (Oxford: Oxford University Press, 2021).

53 Brundage and Shanks, ‘Deaths from Bacterial Pneumonia’, 1198.

54 See for example Thomas Oliver, ‘A Lecture on some of the less common Aspects of Pneumonia: Delivered to the York Medical Society,’ *British Medical Journal*, 1910, 1, 1033–36; M. J. Stewart and H. L. Flint, ‘A Case of Rapidly Fatal Ulcerative Endocarditis due to a Gram-Positive Pleomorphic Diplococcus,’ *Lancet*, 1919, 193, 1114–16; H. T. Chickering and James H. Park, ‘Staphylococcus aureus pneumonia,’ *Journal of the American Medical Association*, 1919, 72, 617–26; James G. Cumming, C. B. Spruit and Ernest J. Aten, ‘Streptococcus pneumonia,’ *Journal of the American Medical Association*, 1919, 72, 704–7.

55 John M. Eyler, ‘The Fog of Research: Influenza Vaccine Trials during the 1918–19 Pandemic,’ *Journal of the History of Medicine and Allied Sciences*, 2009, 64, 401–28, 414.

56 Eyler, ‘The Fog of Research’, 415; G. W. McCoy, V. B. Murray and A. L. Teeter, ‘The Failure of a Bacterial Vaccine as a Prophylactic against Influenza,’ *Journal of the American Medical Association*, 1918, 71, 1997–1997.

57 Yu-Wen Chien, Keith P. Klugman and David M. Morens, ‘Efficacy of Whole-Cell Killed Bacterial Vaccines in Preventing Pneumonia and Death during the 1918 Influenza Pandemic,’ *Journal of Infectious Diseases*, 2010, 202, 1639–48, 1646.

58 T. M. Cherry, ‘Part 1 – Cases Treated at the Various Isolation Hospitals in Melbourne’, Chapter VII in Cumpston, *Influenza and Maritime Quarantine in Australia*, 94. The Rosenow vaccine contained three strains of pneumococci, the strains used for the CSL vaccine are not stated.

59 Museum Victoria, ‘CSL (Commonwealth Serum Laboratories Collection)’, accessed May 28, 2022, https://researchdata.edu.au/csl-commonwealth-serum-laboratories-collection/937367

60 W. J. Penfold, ‘Influenza Vaccine and Inoculation’, Chapter VII in Cumpston, *Influenza and Maritime Quarantine in Australia*, 73–88, 82–83.

61 Eyler, ‘The Fog of Research’, 405.

62 Cherry, ‘Part 1 – Cases Treated at the Various Isolation Hospitals in Melbourne’, 94. See Table 2, Classes 7 & 8.

63 G. Swinden, “The Navy and the 1918-19 Influenza Pandemic - JMVH.” Accessed December 20, 2021. https://jmvh.org/article/the-navy-and-the-1918-19-influenza-pandemic/. The first wave of the pandemic in Australia was delayed as compared to other Western jurisdictions due to prompt quarantine action by Australian authorities. See K. McCracken and P. Curson, ‘Flu Downunder: A Demographic and Geographic Analysis of the 1919 Epidemic in Sydney, Australia,’ Chapter 8 in *The Spanish Influenza Pandemic of 1918-1919* (London: Routledge, 2003), 110–131.

64 Shanks, ‘The “Influenza” Vaccine Used during the Samoan Pandemic of 1918’. See also Francis Grey, ‘Value of Vaccines’, *British Medical Journal*, 1919, 2, 499–500; Francis Grey, ‘Notes on Epidemic Broncho-Pneumonia (Spanish Influenza) in Samoa,’ *Medical Journal of Australia*, 1919, 1, 359–61 and Tom Jefferson and Elian Ferroni, ‘The Spanish Flu through the BMJ’s Eyes,’ *British Medical Journal*, 2009, 339, 1397–99.

65 Armstrong, 1920 *Report of the Director-General of Public Health*, 155.

66 Cherry, ‘Part 1 – Cases Treated at the Various Isolation Hospitals in Melbourne’, Table 4, 112. See also Penfold ‘Influenza Vaccine and Inoculation’, 87. John Oxford and Douglas Gill incorrectly attribute Cherry’s Melbourne Hospital sample to staff of the Victorian Railway Authority. See John S. Oxford and Douglas Gill, ‘Vaccines from the Spanish Influenza as a Firm Foundation for New Developments,’ *Human Vaccines & Immunotherapeutics*, 2020, 16, 2051–55.

67 J. B. Cleland, ‘Part III- Pathological, Bacteriological, and Etiological Investigations into the Influenza Epidemic in Sydney in 1919’, in Armstrong, *Report of the Director-General of Public Health*, 199.

68 Gordon Briscoe, ‘Queensland Aborigines and the Spanish Influenza Pandemic of 1918-1919,’ *Australian Institute of Aboriginal and Torres Strait Islander Studies, Research Discussion Paper*, 1996, 3, 13, 15. The estimated Aboriginal population in Queensland in 1921 was about 17,000 – see Gordon Briscoe, *Counting, Health and Identity: A History of Aboriginal Health and Demography in Western Australia and Queensland, 1900-1940* (Canberra: Aboriginal Studies Press, 2003), Kindle Edition, loc 1334.

69 Briscoe, *Counting, Health and Identity: A History of Aboriginal Health and Demography in Western Australia and Queensland, 1900-1940*, loc 6687-6695. See also Geoffrey W. Rice, ‘How Reminders of the 1918–19 Pandemic Helped Australia and New Zealand Respond to COVID-19,’ *Journal of Global History*, 2020, 15, 421–33, 425.

70 Briscoe, ‘Queensland Aborigines and the Spanish Influenza Pandemic of 1918-1919’, 5.

71 For example see Daniele E. Alves, Svenn-Erik Mamelund, Jessica Dimka et al., ‘Indigenous Peoples and Pandemics,’ *Scandinavian Journal of Public Health*, 2022, 50, (published online).

72 Svenn-Erik Mamelund, Lisa Sattenspiel and Jessica Dimka, ‘Influenza-associated Mortality during the 1918–1919 Influenza Pandemic in Alaska and Labrador: A Comparison,’ *Social Science History*, 2013, 37, 177–29, Table 1, 194–201. For child survivors in Alaska, see Svenn-Erik Mamelund, ‘Geography may explain Adult Mortality from the 1918–20 Influenza Pandemic,’ *Epidemics*, 2011, 3, 46–60.

73 F. T. Cadham, ‘The Use of a Vaccine in the recent Epidemic of Influenza,’ *Journal of the Canadian Medical Association*, 1919, 9, 519–27.

74 John S. Oxford and Douglas Gill, ‘A Possible European Origin of the Spanish Influenza and the First Attempts to Reduce Mortality to Combat Superinfecting Bacteria: An Opinion from a Virologist and a Military Historian,’ *Human Vaccines & Immunotherapeutics*, 2019, 15, 2009–12.

75 ‘To the Medical Profession’, *Daily Telegraph* (Sydney), February 6, 1919, 4.

76 [Advertisement], *Sydney Morning Herald*, February 4, 1919, 6.

77 ‘To the Medical Profession’, *Brisbane Courier* (Brisbane, Queensland), February 10, 1919, 6; ‘Ray, Francois’, *Encyclopaedia of Australian Science*, accessed 18 February, 2022, https://www.eoas.info/biogs/P001206b.htm

78 Biological Institute of Australasia, ‘Pneumonic Influenza Vaccine’, *Sun* (Sydney), April 4, 1919, 7. £1 was about a quarter of the average weekly wage for a male factory worker in Australia in 1920.

79 A Sydney Correspondent, ‘The Doctors’ Harvest,’ *Beaudesert Times* (Beaudesert, Qld), January 24, 1919, 6. This ‘Sydney Correspondent’ seems to have written to a number of newspapers on these lines.

80 W. G. Armstrong, ‘Part I. Epidemiology and Administration, sec. V. Report on the Influenza Epidemic in New South Wales in 1919’, in *Report of the Director-General of Public Health, New South Wales, for the year ended 31st December 1919* (Sydney: Government Printer; 1919), 144–72, 155. For the Victorian campaign, see for example ‘Influenza Epidemic Unabated. Country Cases Reported’, *Weekly Times* (Melbourne), February 15, 1919, 32. For the work of the female Voluntary Aid Detachments, see ‘Activities of the V.A.D’s. Work its own Reward. The Reality of Social Service’, *Northern Star* (Lismore, NSW), April 10, 1919, 7.

81 See for example Chris Stockmann, Krow Ampofo, Andrew T. Pavia et al., ‘Clinical and Epidemiological Evidence of the Red Queen Hypothesis in Pneumococcal Serotype Dynamics,’ *Clinical Infectious Diseases*, 2016, 63, 619–626.

82 Eyler, ‘The Fog of Research’, 406.

83 National Museum of American History, ‘Sherman’s Influenza Bacterial Vaccine Number 38, accessed 14 January, 2022, https://americanhistory.si.edu/collections/search/object/nmah_1071200.

84 Glenvick- Gjonvik Archives, ‘Cutter Laboratory’s Mixed Vaccine for Influenza Pandemic – 1919’, accessed 14 January, 2022, https://www.gjenvick.com/Influenza/CutterLaboratoryMixedVaccineForInfluenza-1918.html

85 The Sherman vaccine was used to a small extent at the US Army’s Camp Dodge in Iowa, but the only published evaluation was that it was thought to be ‘of benefit’. See Edward T. Edgerly, Frank M. Manson, William Dwinell et al., ‘The Influenza-Pneumonia Epidemic at Camp Dodge, Iowa, 1918’, *American Journal of the Medical Sciences (1827-1924)*, 1919, 158, 212–15.

86 World Health Organization, ‘Vaccine Efficacy, Effectiveness and Protection’, accessed 3 April, 2022, https://www.who.int/news-room/feature-stories/detail/vaccine-efficacy-effectiveness-and-protection.

87 Chien et al., ‘Efficacy of Whole-Cell Killed Bacterial Vaccines in Preventing Pneumonia and Death during the 1918 Influenza Pandemic’, 1639.

88 A. H. Ahmed, K. G. Nicholson and J. S. Nguyen-Van-Tam, ‘Reduction in Mortality Associated with Influenza Vaccine during 1989-90 Epidemic’, *Lancet*, 1995, 346, 591–95.

89 Geoffrey A. Weinberg and Peter G. Szilagyi, ‘Vaccine Epidemiology: Efficacy, Effectiveness, and the Translational Research Roadmap’, *Journal of Infectious Diseases*, 2010, 201, 1607–10, 1607.

90 Walter A. Orenstein, Roger H. Bernier, Timothy J. Dondero et al., ‘Field Evaluation of Vaccine Efficacy’, *Bulletin of the World Health Organization*, 1985, 63, 1055–68. For an example of field usage, see G. Lopez-Valencia, T. Renteria-Evangelista, J. de Jesús Williams et al., ‘Field Evaluation of the Protective Efficacy of Mycobacterium Bovis BCG Vaccine against Bovine Tuberculosis’, *Research in Veterinary Science*, 2010, 88, 44–49.

91 Armstrong, *Report of the Director-General of Public Health,* 156.

92
*Ibid.*, 156.

93
*Ibid.*, 108.

94 Chien et al., ‘Efficacy of Whole-Cell Killed Bacterial Vaccines in Preventing Pneumonia and Death during the 1918 Influenza Pandemic’, 1639.

95
*Ibid.*, 1639.

96 Ahmed and Nguyen-Van-Tam, ‘Reduction in Mortality Associated with Influenza Vaccine during 1989-90 Epidemic’, 591.

97 Monica L. Nation, Robert Moss, Matthew J. Spittal et al. ‘Influenza Vaccine Effectiveness against Influenza-Related Mortality in Australian Hospitalized Patients: A Propensity Score Analysis’, *Clinical Infectious Diseases*, 2021, 72, 99–107, 99.

98 Katarzyna Jabłońska, Samuel Aballéa and Mondher Toumi, ‘The Real-life Impact of Vaccination on COVID-19 Mortality in Europe and Israel’, *Public Health*, 2021, 198, 230–37, 230.

99 Penfold, ‘Influenza Vaccine and Inoculation’, 73–88, 82–83.

100 Michael Bresalier, ‘Fighting Flu: Military Pathology, Vaccines, and the Conflicted Identity of the 1918-19 Pandemic in Britain’, *Journal of the History of Medicine and Allied Sciences*, 2013, 68, 87–128, 127.

101 Howard Phillips, ‘The Recent Wave of Spanish’ Flu Historiography’, *Social History of Medicine*, 2014, 27, 789–808.

102 For example Gorsky et al., ‘The 1918/1919 Influenza Pandemic & COVID-19 in Ireland and the UK’, 2021.

103 Cecil and Vaughan, ‘Results of Prophylactic Vaccination against Pneumonia at Camp Wheeler’; Grabenstein and K. P. Klugman, ‘A Century of Pneumococcal Vaccination Research in Humans’.

104 Gary J. Nabel, ‘Designing Tomorrow’s Vaccines’, *New England Journal of Medicine*, 2013, 368, 551–60, 552 (Figure 1B). An influenza vaccine was developed in 1936. For the ‘Golden Age’ see Stanley A. Plotkin, and Susan L. Plotkin, ‘The Development of Vaccines: How the Past Led to the Future,’ *Nature Reviews Microbiology* 9, no. 12 (2011): 889–891, 889.

105 Gupta and Nguyen-Van-Tam, ‘Bacterial Pneumonia and Pandemic Influenza Planning’.

